# Floristic composition and plant community distribution along environmental gradients in Guard dry Afromontane forest of Northwestern Ethiopia

**DOI:** 10.1186/s12862-023-02154-6

**Published:** 2023-08-28

**Authors:** Yitayih Dagne, Liyew Birhanu

**Affiliations:** Department of Biology, Debre Markos University, P.O. Box: 269, Debre Markos, Ethiopia

**Keywords:** Community, Diversity index, Endemic, Floristic composition

## Abstract

**Background:**

Dry evergreen montane forests of Ethiopia provide economic and ecological services for the community but it is under several threats of natural and anthropogenic disturbances. The study aimed to investigate the floristic composition, species diversity, and plant community distribution of Guard forest along environmental gradients.

**Methods:**

A systematic sampling technique was used to collect vegetation and environmental data. Fifty eight plots each with 400 m^2^ (20 m X 20 m) were established for trees and shrubs and 2 m x2m (4 m^2^) for herbs along eleven transect lines. Shannon Weiner diversity index and evenness were used to assess the species diversity and richness. The similarities among plant communities were computed using Sorenson’s similarity index. The plant community types and vegetation-environment relationships were analyzed using hierarchical cluster analysis and Canonical correspondence analysis (CCA) in R software, respectively.

**Results:**

A total of 137 plant species belonging to 111 genera and 55 families were identified. The most dominant families in the study area were Fabaceae and Asteraceae. Among the total plant species documented in the forest, 42(30.65%) were trees, 36 (26.28%) were shrubs, 48(35.04%) were herbs, and 11 (8.03%) were climbers. Of the total species, 14(10.22%) species are endemic to Ethiopia and Eritrea. Three plant community types were identified by cluster analysis. The Shannon-Weiner Diversity Index was 3.39 and evenness was 0.87 for the forest. The pattern of plant species distribution was significantly influenced by altitude, pH, BD, slope, and charcoal (*P* < 0.05).

**Conclusion:**

Guard forest has good species diversity and richness, and supports different endemic plant species that show the potential of the area to support useful but some of the characteristics species are not found in the forest and others are rare in their existence due to the presence of disturbances and need immediate conservation to ensure sustainable use and management of the forest.

**Supplementary Information:**

The online version contains supplementary material available at 10.1186/s12862-023-02154-6.

## Background

Ethiopia is a land-locked country located in the Northeastern part of the African continent between 3^o^24' to 14º 53´N and 33º00' to 48^o^00'E and covers a land surface area of 1,104,300 km^2^(Friis et al*.*, 2010); with a broad latitudinal and altitudinal range from 116 m below sea level to 4,620 m above sea level [1, 2]. This altitudinal variation of the country helps for the emergence of wide range of habitats that are suitable for the evolution and survival of various plant and animal species, making the country better for the endemism of plant and animal species and overall biodiversity of the country [3, 4]. Approximately. there are about 6,027 vascular plant species with 647 (10.74%) endemism in the Flora of Ethiopia and Eritrea of which 3,875 species in Ethiopia only, 270 in Eritrea only and 1,882 common both countries [5].

Dry Afromontane forests of Ethiopia are the most degraded and fragmented forest ecosystem in the world due to the increase in human population in the area because the Afromontane area has more suitable weather conditions for human settlement than other ecosystems that exploit the forest for getting agricultural lands, settlement, tree harvesting for different materials, grazing purposes, infrastructure development, and urbanization. In Dry Afromontane forests, the persistence of the remnant forest patches and their indigenous species in many areas are threatened [6].

Anthropogenic disturbance severely affects the diversity, composition, structure, and regeneration status of forests [7]. The degradation of forests in Ethiopia is closely linked to the ongoing population growth [8]. The major cause for plant loss is the increasing human population that creates pressure to destroy natural habitats for expanding agriculture, expanding urbanization, firewood, getting charcoal, settlement, herbal medicines, and other resources got from plants [9] According to [8], around 83% of endangered plant species loss is primarily caused by human activities.

Different study has been made in Amhara regional state related to the floristic composition of the forest such as [10, 11] but there is limited study in Dejen district related to floristic composition and plant community distribution along environmental gradients.

## Methods

### Study area

**Fig. 1:**
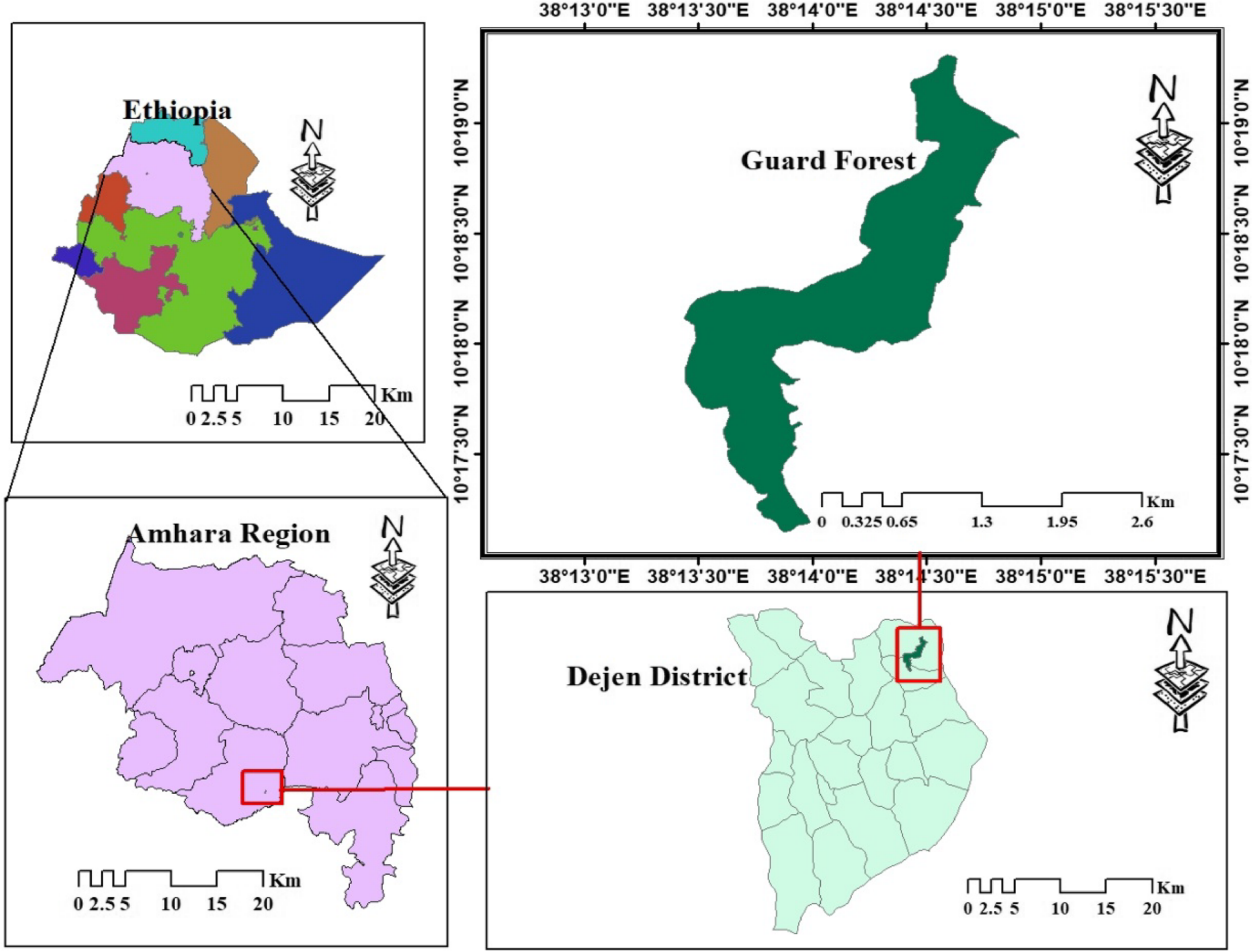
Map of Ethiopia, Amhara region, Dejen district and Guard forest

The study was conducted in Guard Forest which is located in Kokuha kosekos kebele of Dejen district, East Gojjam Zone, Amhara National Regional State, which is situated between 10° 17'N to 10° 19'N and 38°14'E to 38°15'E with the altitudinal range between 2019 and 2230 m a.s.l. (Fig. 1). It’s approximately 40 km northeast of the district town and the total area of forest covers 28 ha. The geology of the study site is characterized by nithosols soil types in the northwestern, southwestern, northern, and central parts of the district. In some midland parts of the district, there is black soil that displays cracks and sticky characteristics during dry and wet seasons, respectively and soils that are found in lowland areas are gray, red, and whitish which are shallow and extremely stony [12].

**Fig. 2:**
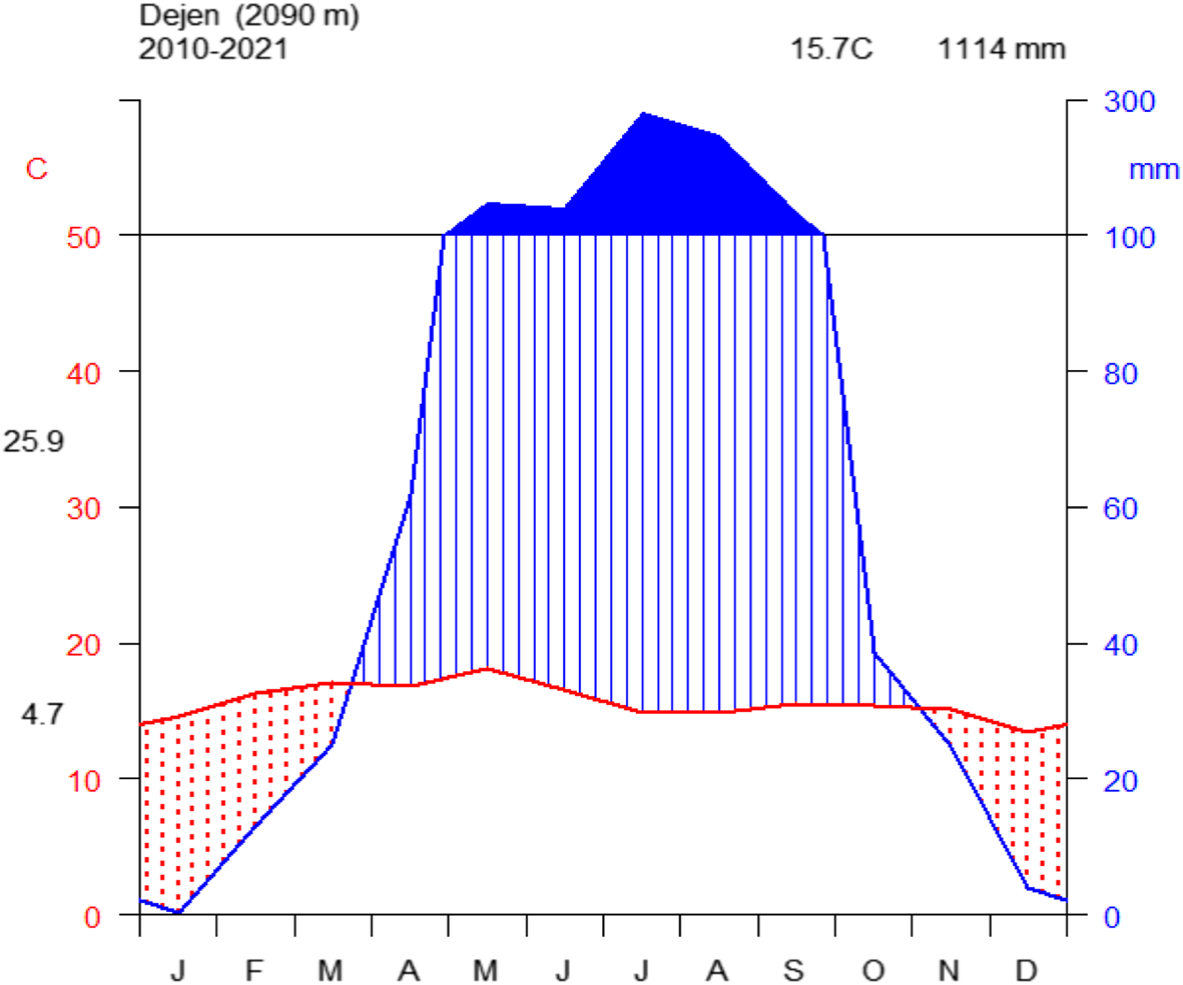
Climadiagram of Dejen **(**Data source: National Meteorological Agency, 2022)

Rainfall and temperature data for the study area were obtained from National Meteorology Service Center, Bahir Dar, for twelve years (2010 – 2021). The analysis of the obtained data shows that the mean minimum temperature is 4.7^0^C and the mean maximum temperature is 25.9 ^0^C, while the mean annual temperature of the study area is 15.7 ^0^C. The hottest month is March With a mean maximum temperature of 25.9◦c and the coldest month is December with a minimum mean temperature of 4.7^0^C. The mean annual rainfall value for the study area was 98.74 mm which varied greatly from season to season. Generally, the study area has low rainfall from December to February and the main rainy season is June to August (Fig. 2).

Dry Evergreen Afromontane Forest vegetation type occurs in an altitudinal range of 1800 to 3000 m a.s.l [2]. Guard forest has an altitudinal range from 2019 m a.s.l. to 2230 m a.s.l and its plant composition is a mixture of *Carissa spinarum*, *Grewia ferruginea, Olea europea* subsp. *cuspidata, Rosa abyssinica*, *Vachellia abyssinica, Calpurena aurea, Croton macrostachyus, Milletea fergunea, Clutia abyssinica.* This is a good indication that Guard forest vegetation type belongs to dry evergreen Afromontane Forest.

### Sampling technique

A systematic sampling technique was used for vegetation and environmental data collection. Eleven line transects were laid vertically with a distance of 200 m between each line transect. The first line transect begins from the starting side of the forest and the first plot was laid 50 m away from the cultivated land starting from the base to the top of the forest and the distance between each plot is 100 m. When the distance of 100 m lay on the hill, bare land, or large stone, the plot was laid 5 m below or above the point of the measured distance of plots. The number of plots per transect varies depending on the length of each transect and a total of 58 plots were collected from the forest. Each plot has 20 m × 20 m (400 m^2^) for trees and shrubs and from the four corners and the center of the main sample plot subplot which has 2 m × 2 m (4 m^2^) were laid for herbs [13]. A complete list of trees, shrubs, and herbs including epiphytes was taken from systematically selected plots along each transect.

Finally, the cover abundance of all woody plant species was estimated in percentage and converted following the Braun-Blanquet scale from 1 to 9 [14]. The local names of all species were recorded during fieldwork and included in the list of taxa and specimens of all plant taxa were collected, pressed, dried, and brought to Debre Markos University herbarium room for identification. Their families, genera, and scientific names were identified using the published Flora of Ethiopia and Eritrea from volumes 1- 8.

Environmental data such as soil samples, slope, latitude, longitude, and altitude were collected from each plot in which vegetation data were collected. The soil samples were collected from the four corners and center of the main plot and mixed to produce a composite sample with depth of 30 cm. From each plot, 0.5 kg of soil sample was taken. The latitude, longitude, and altitude were taken from the center of each main plot measured using GPS and the slope of the area was collected from the edge of the main plot using a clinometer. All collected soil samples which have collected from all plots were brought to Debre Markos University Soil Sciences lab room for testing soil size distribution, bulk density, particle density, soil moisture content, pH, electric conductivity, and organic matter.

To determine the moisture content of soil, the soil samples were air-dried and then weighed before they were oven-dried at 105 °C for 24 h and cooled in a desiccator, and weighed after being dried in an oven. The estimation of soil moisture content percentage was calculated by the following equation [15].

The bulk density of soil was analyzed by calculating the ratio of oven-dried soil weight to the volume of the soil using a Graduate measured volume of soil.

$$\mathrm{BD}=\frac{\mathrm{Mass\;of\;oven}-\mathrm{dry\;soil}}{Volume\;of\;soil}$$

where: BD = bulk density.

Soil pH was measured by preparing a 1:2.5 soil to water suspension and measuring soil PH using a glass electrode pH meter. The electrical conductivity of the soil sample was measured by preparing 1 soil with 2.5 water suspensions and measuring using a conductivity meter, Soil texture tests to determine the soil whether sand soil; silt soil or clay soil were analyzed by Bouycous Hydrometer method [15]. To do the test Air-dried soil sample was passed through a sieve, 50 g of air-dried sieve soil sample and dispersing agent, 5 g of sodium hexametaphosphate were separately weighed, 1000 ml of pure water was measured with a graduated cylinder and mixed with measured sodium hexametaphosphate then sieve soil sample was added and mix vigorously for about 3 min and Hydrometer was inserted into the mixture after 45 s, 2 h. Hydrometer readings were recorded to calculate the percentage of sand and clay, respectively. After the percentage of sand, clay, and silt was calculated, soil texture class was determined using USDA Soil Textural Triangle.

The total organic matter content of the soil was determined by ignition, which involves combustion of soil samples at high temperatures using a Furnace at 550C° for 3 h and measuring weight loss after ignition [17]. The soil samples were air-dried and then oven-dried at 105 °C for 24 h and cooled in a desiccator, then weighed were measured before combusted at 550 °C for 3 h in the furnace after combustion, the samples were cooled in a desiccator and weighed again [18].

Human disturbance for firewood, charcoal, timber, grazing, and other data was estimated from each main plot. Human interferences were recorded based on the presence or absence of stumps, logs, grazing intensity, and signs of fuel wood collection. The grazing intensity of the forest was estimated based on visual observation of different symptoms of livestock effects such as dung droppings and herbage cuttings. The types of disturbances were arranged qualitatively by observing their remaining parts on each plot. All types of disturbances were ranked into relatively absent (no disturbance), which score 0, low (1), medium (2), and high (3)for each level of disturbance [16]. The sum of all scores for each plot provides an overall ranking of anthropogenic disturbances in each community. High ranks signify high levels of anthropogenic disturbance and low ranks reveal low levels of disturbance.

### Data analysis

**Fig. 3:**
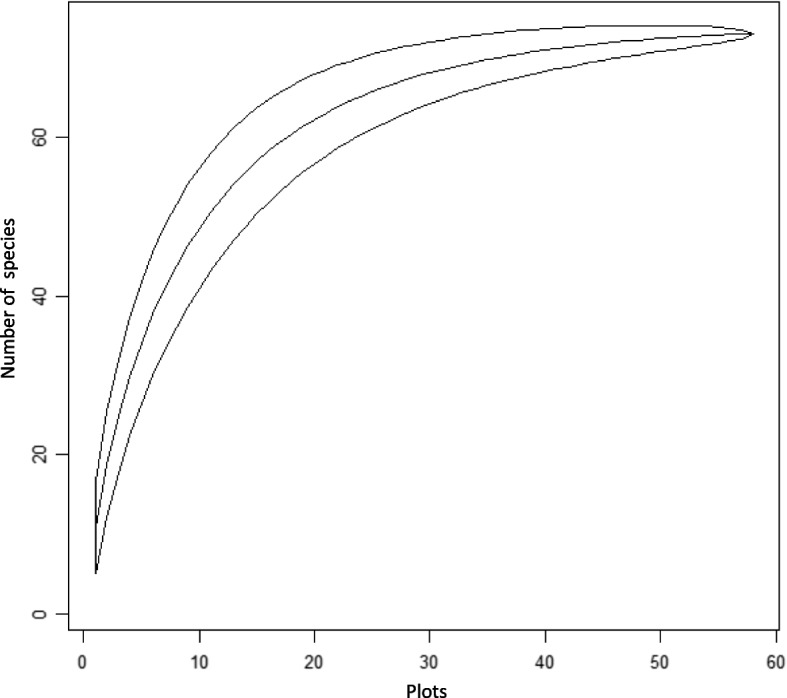
Species Accumulation Curve of Guard forest

A species accumulation curve (SAC) of the data was drawn to ensure that enough plots were collected from the study area using library (vegan)” package in R software program (vers 4.1.2). SAC of Guard forest shows that the graph rises dramatically in the first 20 plots and there is a gradual increment between plots 20 and 50 and constantly moves above plot 50, this indicates that the probability of getting new species is less and the sample plot is enough to represent the forest (Fig. 3). Prior to the cluster, ordination and one way ANOVA exploratory data analyses, including a correlation matrix and an assessment of normality using Shapiro Wilk test, were used to examine the appropriateness of environmental variables and vegetion data. These analyses guided pre processing of the data and informed the type of cluster analyses, ordination and one way ANOVA used.

**Fig. 4:**
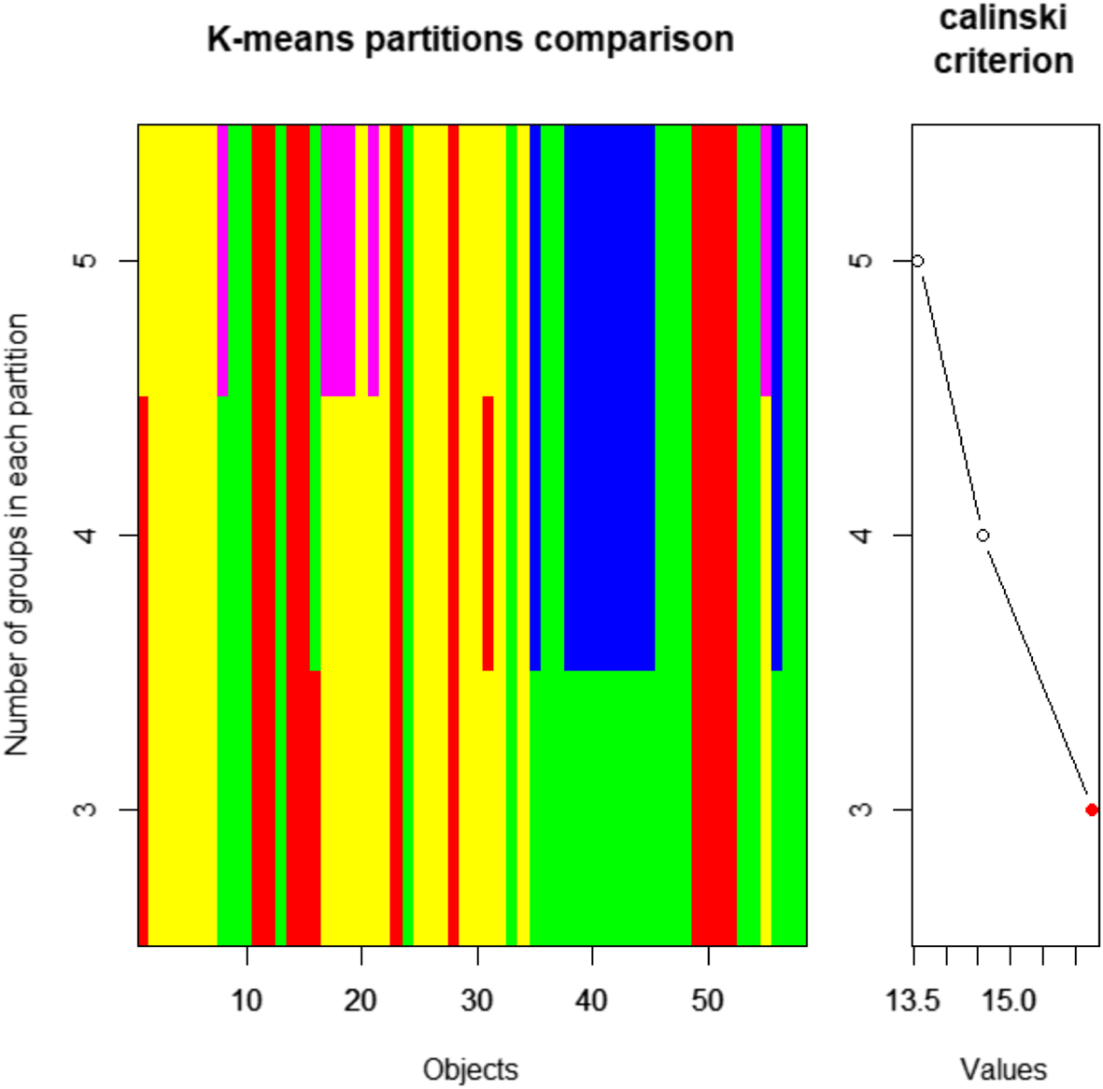
K-means cascade plot showing the group attributed to each object for each partition. The plot shows the group attributed to each object for each partition (rows of the graph). The rows of the graph are the different values of k. The groups are represented by different colours; there are three colours for k = 3, four colours for k = 4, and so on. Another graph shows the values of of the criterion ( calinski) for determining the best partition

Hierarchical cluster analysis is one of the most commonly used multivariate techniques to analyze the community of ecological data using R software (Ver 4.1.2). It helps to group a set of vegetation samples based on their floristic similarities. In this study, agglomerative hierarchical classification using similarity ratio and Ward method (Minimum-variance clustering) was performed to classify the vegetation into plant community types based on the abundance data of the species in each plot. The decision on the number of groups (clusters) was based on objective methods of obtaining an optimal number of clusters (K), we used K-means partitioning using cascadeKM (calinski) and Similarity Ratio with library vegan in R program. The Calinski-Harabasz criterion is sometimes called the variance ratio criterion (VRC). The optimal number of clusters corresponds to the solution with the highest Calinski-Harabasz index value. In the Calinski Criterion, several partitions from K-means clustering are created forming a cascade from a small to a large number of groups. The maximum value of these indices/partitions is supposed to indicate the best partition. The Calinski-Harabasz criterion recovered the correct number of groups the most often. The function cascadeKM produced two plots. The graph on the left has the number of groups and represented by different colors indicate the clusters at each successive division. The graph on the right shows the values of the criterion ( calinski or ssi) for determining the best partition. The highest value of the criterion is marked in red and was used to identify the appropriate number of clusters. There was a maximum value partitions at k of 3, indicating that this was the optimal number of clusters (Fig. 4). Moreover, Simple visual observation suggestes three clusters at the height of 1.5 of the dendrogram.

The species diversity of each forest community was calculated using the Shannon–Wiener diversity index [13].

$$\mathrm H'=-\sum\nolimits_{i=1}^spi\ln pi$$

where: H' = Shannon diversity index; **s** = number of species, Pi = proportion of individuals or abundance of the i^th^ species expressed as a proportion of total cover in the sample; and $ln$ = the natural logarithm.

Evenness was calculated by the Shannon evenness index (E), which measures the association between species and the level of similarity between samples.

$$\mathrm E=\frac{H'}{H_{max}}=\frac{-\sum_i^spilnpi}{\mathit{ln}s}$$

Sorensen’s similarity index was used to evaluate species composition and species distribution among all plant communities of the forest vegetation following [13].

$$\mathrm{Ss}=\frac{2a}{(2a+b+c)}$$

$$\mathrm{\% Ss}=\frac{2a}{(2a+b+c)}\mathrm{ x}100$$

where: Ss = Sorensen’s similarity coefficient; b = number of species in community 1; c = number of species in community 2; a = number of species common to both communities 1 and 2.

Ordination is a multivariate method that expresses the relationships between sample species and environmental variables in a low-dimensional space using ordination diagrams [19]. To select the best ordination technique, it was necessary to determine the assemblage variation (gradient length), so detrended correspondence analysis (DCA) was performed. If the gradient length is 3 or more, the assemblage variation is over a larger range, and the unimodal-based approach of CA or CCA is appropriate [20]. The relationship of anthropogenic variables, soil type, slope, and altitudinal variables with forest structure was analyzed using the multivariate ordination technique of Canonical correspondence analysis (CCA) due to its heterogeneity and visualization of the graph. Adonis test was performed to determine the significance of the environmental variables on plant community distribution. One-way ANOVA was used whether there were significant mean differences among plant communities about anthropgenic disterbances.

## Results

### Floristic composition of the forest

A total of 137 plant species were collected from Guard Forest patch, which is belonging to 111 genera and 55 families. Out of these species, 130 were collected from 58 plots, and the remaining 7 species, namely, *Withania somnifera*, *Securidaca longepedunculata*, *Combretum molle, Ximenia americana, Zizyphus mucronata*, *Salix mucronata,* and *Gossypium hirsutum* were collected outside the plots but found within the boundary of forest around sample plot up to 10 m distances. The complete list of plant species gathered in the study area was shown in (Table 1). The most dominant families of the study area were Fabaceae, which are represented by 20 species (15%) and followed by Asteraceae, which contain 16 species (12%) and Lamiaceae, represented by 7 species (5%) of the total floristic composition.

**Table 1:** The list of plant species in Guard forest

No.	Scientific name	Family	Habit	Local name
1.	*Abutilon longicuspe* Hochst. ex A. Rich	Malvaceae	Shrub	Nachachela
2.	*Acanthus polystachyous* Delile	Acanthaceae	Shrub	Kosheshila
3.	*Acanthus sennii* Chiov	Acanthaceae	Shrub	Kosheshila
4.	*Achyranthes aspera* L	Amaranthaceae	Herb	Telenji
5.	*Acokanthera schimperi* (A.DC.) Schweinf	Apocynaceae	Tree	Merth
6.	*Agave sisalana* Perro exEng	Agavaceae	Shrub	Cherat
7.	*Ageratum conyzoides* L	Asteraceae	Herb	Arem
8.	*Albizia amara* sub sp *amara* (Roxb.) B.Boivin	Fabaceae	Tree	Shifere
9.	*Albizia schimperiana* Oliv	Fabaceae	Tree	Sesa
10.	*Aloe macrocarpa* Todaro	Aloeaceae	Herb	Eret
11.	*Andropogon abyssinicus* Fresen	Poaceae	Herb	Gajja
12.	*Anethum graveolens* L	Apiaceae	Herb	Enselale
13.	*Arundo donax* L	Poaceae	Herb	Shembeko
14.	*Bersama abyssinica* Fresen	Melianthaceae	Tree	Azamera
15.	*Bidens macroptera (*Sch.-Bip.)	Asteraceae	Herb	Adey-Abeba
16.	*Bidens pilosa* L	Asteraceae	Herb	Ysatan-Merfy
17.	*Brucea antidysenterica* J.F. Mill	Simaroubaceae	Shrub	Wagnos
18.	*Buddleja polystachya* Fresen	Loganiaceae	Tree	Anfar
19.	*Caesalpina decapetala* (Roth) Alston	Fabaceae	Climber	Konter
20.	*Calpurnia aurea* (Ait.) Benth	Fabaceae	Shrub	Digeta
21.	*Capparis tomentosa* Lam	Capparidaceae	Climber	Gumero
22.	*Carissa spinarum* L	Apocynaceae	Shrub	Agam
23.	*Celtis africana* Burm. F	Ulmaceae	Tree	Kawoot
24.	*Cirsium schimperi* (Vatke) Cuf	Asteraceae	Herb	Kosendro
25.	*Cissus quadrangularis* L	Vitaceae	Climber	Gebezan
26.	*Clematis simensis* Fresen	Ranunclaceae	Climber	Azohareg
27.	*Clerodendrum indicum* L	Lamiaceae	Shrub	Misrch
28.	*Clutia abyssinica* Jaub. & Spach	Euphorbiaceae	Shrub	Fiyele-Fej
29.	*Combretum collinum* Fres*en*	Combretaceae	Tree	Zegudem
30.	*Combretum molle* R. Br. ex G. Don	Combretaceae	Tree	Abalo
31.	*Commelina benghalensis* L	Commelinaceae	Herb	Yewha- Aqur
32.	*Commiphora africana* A. Rich	Burseraceae	Tree	Anqa
33.	*Cordia africana* Lam	Boraginaceae	Tree	Wanza
34.	*Cotula abyssinica* Sch.Bip.ex A.Rich	Asteraceae	Herb	-
35.	*Crassocephalum sarcobasis* (DC.) S.Moor	Asteraceae	Herb	-
36.	*Crateva adansonii* DC	Capparidaceae	Tree	Dingay-Seber
37.	*Crepis rueppellii* Sch.Bip	Asteraceae	Herb	Yefyel-Wetet
38.	*Croton macrostachyus* Del	Euphorbiaceae	Tree	Bisana
39.	*Cucumis ficifolius* A. Rich	Cucurbitaceae	Climber	Yemdir-Emboy
40.	*Cynodon dactylon* (L.) Pers	Poaceae	Herb	Serdo
41.	*Cynoglossum coeruleum* Hochest ex.A.DC.in	Boraginaceae	Herb	Shungeg
42.	*Cyperus longus* L	Poaceae	Herb	Engicha
43.	*Cyphostemma adenocaule* Stued.ex A.Rich	Vitaceae	Climber	Mariam-Meknet
44.	*Datura stramonium L*	Solanaceae	Herb	Astenager
45.	*Dichrostachys cinerea* (L.) Wight &Arn	Fabaceae	Tree	Ader
46.	*Dodonaea viscosa* L	Sapindaceae	Tree	Kitketa
47.	*Dombeya torrida* (J.F.Gmel.) P. Bamps	Sterculiaceae	Tree	Wolkefa
48.	*Dracaena steudneri* Engl	Dracaenaceae	Herb	Merko
49.	*Ekebergia capensis* Sparrm	Meliaceae	Tree	Sembo
50.	*Erythrina abyssinica* Lam. ex DC	Fabaceae	Tree	Bogert /Korch/
51.	*Euclea racemosa (*A. DC.) White	Ebenaceae	Tree/shrub	Dedeho
52.	*Euphorbia tirucalli* L	Euphorbiaceae	Shrub	Kincheb
53.	*Euphorbium candelabrum* Trem. ex Kotschy	Euphorbiaceae	Tree	Kulkual Adem
54.	*Ferula communis* L	Apiaceae	Herb	Dog
55.	*Ficus sur* Forssk	Moraceae	Tree	Shola
56.	*Ficus sycomorus* L	Moraceae	Tree	Abar_Worka
57.	*Ficus thonningii* Blume	Moraceae	Tree	Chebha
58.	*Ficus vasta* Forssk	Moraceae	Tree	Warka
59.	*Galinsoga parviflora* Cav. I	Asteraceae	Herb	Nekay
60.	*Girardinia bullosa* (Steudel) wedd	Urticaceae	Herb	Dobi
61.	*Gossypium hirsutum* L	Malvaceae	Shrub	Tete
62.	*Grewia bicolor* Juss	Tiliaceae	Tree	Sofa
63.	*Grewia ferruginea* Hochst. ex A.Rich	Tiliaceae	Tree	Lenquata
64.	*Guizotia scabra* (Vis.) Chiov	Asteraceae	Herb	Mech
65.	*Hygrophila auriculata* K. Schum	Acanthaceae	Herb	Amekala
66.	*Hyparrhenia anthistirioides* (Hochst.ex.A.Rich.). Stapf	Poaceae	Herb	Senblet
67.	*Impatiens rothill* hook.f	Balsaminaceae	Herb	Engshret
68.	*Jusminum grandifolum* L	Oleaceae	Herb	Tembelel
69.	*Justicia schimperiana* Hochst. ex Nees	Acanthaceae	Shrub	Sensel
70.	*Kalanchoe petitiana* A.Rich	Crassulaceae	Herb	Yekola Enjera
71.	*Laggera crispate* (Vahl) Hepper	Asteraceae	Herb	Kes-Bedaje
72.	*Laggera tomentosa* Sch. Bip. Ex A.rich	Asteraceae	Herb	Kes-Keso
73.	*Leucas martinicensis* (Jacq.) R. Br	Lamiaceae	Herb	Ras-Kimr
74.	*Lippia adoensis* Hochst. ex Walp	Verbenaceae	Shrub	Kese
75.	*Maesa lanceolata* Forssk	Myrsinaceae	Tree	Ashkamo
76.	*Maytenus arbutifolia* (A. Rich.) Wilczek	Celastraceae	Shrub	Atate
77.	*Maytenus senegalensis* (Lam)	Celastraceae	Shrub	Nech Atate
78.	*Medicago polymorpha* L	Fabaceae	Herb	Wajema
79.	*Millettia ferruginea* (H*ochst.)* Bak	Fabaceae	Tree	Birbira
80.	*Mimusops kummel* A. DC	Sapotaceae	Tree	Shiye
81.	*Momordica foetida* Schumach	Cucurbitaceae	Climber	Yamora –Kelb
82.	*Nicandra physalodes* L	Solanaceae	Herb	Ate-Faris
83.	*Notholaena standleyi* Alan R. Smith	Aspleniaceae	Herb	Sarenst
84.	*Ocimum lamiifolium* Hochst. ex Beth	Lamiaceae	Shrub	Dama-Kese
85.	*Oplismenus hirtellus* (L.)	Poaceae	Herb	Yekok-Sar
86.	*Opuntia ficus-indica* L	Cactaceae	Tree	Kulkual
87.	*Orobanche minor* (smith)	Orobanchaceae	Herb	Sar-Nekas
88.	*Osyris quadripartite* Decn	Santalaceae	Shrub	Keret
89.	*Otostegia integrifolia* Benth	Lamiaceae	Shrub	Tunjit
90.	*Pelargonium multibracteatum* Hochst. ex A.Rich	Geraniaceae	Herb	Ejamsele
91.	*Phragmanthera regularis* (Sprague) M.Gilbert	Loranthaceae	Shrub	Tegadera
92.	*Phytolacca dodecandra* L 'Herit	Phytolaccaceae	Climber	Endode
93.	*Plectranthus bellus* P.I.Forst	Lamiaceae	Shrub	Bosbose
94.	*Plectranthus ornatgus* Hochst. ex Giirke	Lamiaceae	Herb	
95.	*Plumbago zeylanica* L	Plumbaceae	Shrub	Amera
96.	*Premna schimperi* Engl	Verbenaceae	Shrub	Checho
97.	*Rhamnus staddo* A. Rich	Rhamnaceae	Shrub	Yedur Gesho
98.	*Rhus glutinosa* A*. Rich*	Anacardiaceae	Tree	Ambus
99.	*Rhus retinorrhoea* Steud. ex Oliv	Anacardiaceae	Tree	Telom
100.	*Rhus vulgaris* Meikle	Anacardiaceae	Tree	Ashqamo
101.	*Ricinus communis* L	Euphorbiaceae	Shrub	Chakma
102.	*Rosa abyssinica* Lindley	Rosaceae	Climber	Qega
103.	*Rumex abyssinicus* Jacq	Polygonaceae	Herb	Mekmeko
104.	*Rumex nepalensls* Spreng	Polygonaceae	Herb	Tult
105.	*Rumex nervosus* Vahl	Polygonaceae	Shrub	Embuate
106.	*Salix mucronata* Thumb	Salicaceae	Shrub	Kiya
107.	*Salvia tiliifolia* Vahl	Lamiaceae	Herb	Yewsha- Zekakebe
108.	*Schefflera abyssinica* (Hochst. ex A.Rich.)	Araliaceae	Tree	Getama
109.	*Securidaca longepedunculata* Fresen	Polygalaceae	Shrub	Tsemnahe
110.	*Senna didymobotrya* Fresen	Fabaceae	Shrub	Gim Abeba
111.	*Senna septemtrionalis* (Viv.) Irwin & Bameby	Fabaceae	Shrub	Gufa mesel
112.	*Senna singueana* (Del.) Lock	Fabaceae	Shrub	Gufa
113.	*Sesbania sesban* (L.) Merr	Fabaceae	Shrub	Sespania
114.	*Solanecio gigas* (Vatke) C. Jeffrey	Asteraceae	Shrub	Boze
115.	*Solanum indicum* L	Solanaceae	Shrub	Embuay
116.	*Solanum marginatum* L	Solanaceae	Shrub	Embuay
117.	*Solanum nigrum* L	Solanaceae	Herb	Awote
118.	*Stephania abyssinica* Dillon & A. Rich	Menispermaceae	Climber	Yayit-Gero
119.	*Stereospermum kunthianum* Cham	Bignoniaceae	Tree	Washte
120.	*Tagetes minuta* L	Asteraceae	Herb	Dej-admik
121.	*Trifolium decorum* Chiov	Fabaceae	Herb	Maget
122.	*Trifolium quartinianum* A.Rich	Fabaceae	Herb	Mget
123.	*Trifolium schimperi* A. Rich	Fabaceae	Herb	Maget
124.	*Triumfetta pilosa* Roth	Tiliaceae	Herb	Shciget
125.	*Urtica simensis* Steudel	Urticaceae	Herb	Sama
126.	*Vachellia brevispica* Harms	Fabaceae	Shrub	Kentafa
127.	*Vachellia bussei* (Harms ex Y.Sjöstedt)	Fabaceae	Tree	Girare
128.	*Vachellia polyacantha* Hochst. ex A. Rich	Fabaceae	Tree	Qento
129.	*Vachellia seyal* Del	Fabaceae	Tree	Nech_Girar
130.	*Vachellia sieberiana* DC	Fabaceae	Tree	Dera
131.	*Vernonia leopoldii* Sch. Bip. ex Walp	Asteraceae	Shrub	Chibo
132.	*Vernonia amygdalina* Del	Asteraceae	Tree	Girawa
133.	*Withania somnifera* L	Solanaceae	Shrub	Gizewa
134.	*Xanthium strumarium* L	Asteraceae	Herb	Gama-Tebtb
135.	*Ximenia Americana L*	Oleaceae	Tree	Enkoy
136.	*Zehneria scabra* L	Cucurbitaceae	Climber	Hareg- Resa
137.	*Zizyphus mucronata* Wild	Rhamnaceae	Tree	Foch

In the forest, different species have different growth forms such as trees; shrubs, herbs, and climbers were included. Herbs occupied the highest proportion followed by trees, shrubs, and climbers. Most commonly**,** existing tree species of the forest were *Croton macrostachyus, Millettia ferruginea, Rhus glutinosa, Celtis africana, Grewia bicolor,Albizia schimperiana;* Common shrub species include *Calpurnia aurea, Euclea racemos, Maytenus senegalensis, Senna didymobotrya,Premna schimperi, Plumbago zeylanica, Carissa spinarum;* Common herb species include *Achyranthes aspera, Salvia tiliifolia, Tagetes minuta, Urtica simensis, Xanthium strumarium;*and *Cissus quadrangularis*, *Phytolacca dodecandra, Capparis tomentosa, Clematis simensis, Cyphostemma adenocaule, Zehneria scabra* are commonly existing climber species of Guard forest.

Of the total of 137 plant species composition of Guard forest, 14 species (10.22%) are endemic to Ethiopia and Eritrea. These species are grouped into 10 families and 14 genera. Of the total of endemic species, 2 Species are trees, 7 Shrubs, and 5 herbs. Depending on the IUCN Criteria for the level of threatened species, 9 species are least concern (LC), 3 species have been considered as near threatened (NT), and 2 species are vulnerable (Table 2).

**Table 2:** Endemic species and their IUCN category **(** LC, Least Concern; NT, Near threated; VU, Vulnerable)

No.	Scientific Name	Family	Habit	IUCN category
1.	*Acanthus sennii*	Acanthaceae	Shrub	NT
2.	*Impatiens rothill*	Balsaminaceae	Herb	LC
3.	*Kalanchoe petitiana*	Crassulaceae	Herb	LC
4.	*Laggera tomentosa*	Asteraceae	Herb	NT
5.	*Lippia adoensis*	Verbenaceae	Shrub	LC
6.	*Millettia ferruginea*	Fabaceae	Tree	LC
7.	*Rhus glutinosa*	Anacardiaceae	Tree	VU
8.	*Solanecio gigas*	Asteraceae	shrub	LC
9.	*Solanum marginatum*	Solanaceae	Shrub	LC
10.	*Trifolium decorum*	Fabaceae	Herb	NT
11.	*Urtica simensis*	Urticaceae	Herb	LC
12.	*Vernonia leopoldii*	Asteraceae	Shrub	LC
13.	*Cirsium schimperi*	Asteraceae	Herb	LC
14.	*Clutia abyssinica*	Euphorbiaceae	Shrub	Vu

The Shannon-Weiner Diversity Index of Guard forest varied from 3.03 to 3.82 and the evenness value ranges from 0.81 to 0.92, and the species richness was between 32 and 65 (Table 3). The Sorensen’s similarity coefficient of Guard forest shows that community 1 and community 3 have the highest similarity (45%), followed by community 2 and community 3(37%), and community 1 and community 2(36%). A relatively low similarity ratio was observed between community 1 and community 2 (Table 4).

**Table 3:** Species richness, evenness and diversity indices of plant community type

Community Types	Altitudinal range	Richness	Diversity (H')	Evenness (E)
1	2032–2205	65	3.82	0.92
2	2030–2133	59	3.32	0.81
3	2052–2211	32	3.03	0.87

**Table 4:** Pair wise comparison of Sorensen’s similarity coefficient between plant communities

Community types	1	2	3
1			
2	0.36		
3	0.45	0.37	

### Community types and characteristics of species

**Fig. 5:**
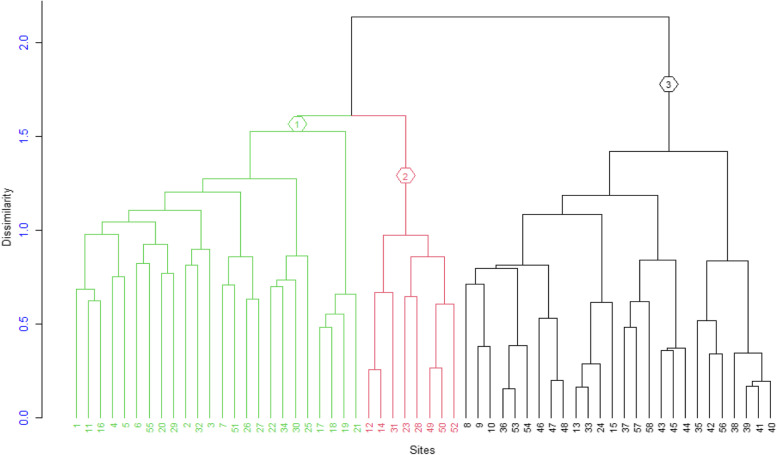
Dendrogram of the vegetation data obtained from hierarchical cluster analysis

Of the total of 137 plant species, 73 woody plant species were used for vegetation classification with the help of cluster analysis using the R software program (vers 4.1.2). Three plant community types were identified ( Fig. 5). The community is named after one or two dominant indicator tree or shrub species selected by the relative magnitude of their synoptic values (Table 5).

**Table 5:** Synoptic values of characteristics species in each community type (C1 – C3)

Species	Synoptic value		
	**C1**	**C2**	**C3**
*Millettia ferruginea*	**2**	0.75	0
*Senna didymobotrya*	**1.88**	0	1.08
*Stereospermum kunthianum*	1.53	**6**	3.88
*Croton macrostachyus*	0.33	**4.12**	0
*Rhus retinorrhoea*	0.62	**4**	0.65
*Euclea racemosa*	1.12	1.12	**5.81**
*Maytenus senegalensis*	1.38	0	**5.23**

### Community 1, *Millettia ferruginea—Senna didymobotrya* community type

This community type was represented by 24 plots and 65 species. This community was named after *Millettia ferruginea* and *Senna didymobotrya* dominating the area because of their relatively high synoptic value. This community is distributed at altitudes between 2032 and 2205 m.a.s.l. with a mean altitude of 2098 m a.s.l. Dominant Woody species associated with this community are *Millettia ferruginea, Albizia schimperiana, Celtis africana, Erythrina abyssinica, Ficus thonningii, Osyris quadripartite, Ficus vasta, Grewia bicolor, Buddleja polystachya, Senna singueana, Calpurnia aurea, Premna schimperi, Croton macrostachyus, Rhus glutinosa, Cissus quadrangularis, Senna didymobotrya, Maytenus senegalensis, Grewia ferruginea, Phytolacca dodecandra, and Maytenus arbutifolia and Euclea racemosa.* This community type has better vegetation cover than other community types of forest and the most representative plots of this community are found relatively far from anthropogenic disturbances such as agricultural expansion, firewood, and grazing areas.

### Community 2, *Stereospermum kunthianum—Croton macrostachyus* community type

This community lies between the altitudinal range of 2052 and 2211 m a.s.l., contains 8 plots, and represented by 32 species. This community was named Because of the high synoptic value of *Stereospermum kunthianum* and *Croton macrostachyus*. Dominant Woody species associated with this community are *Stereospermum kunthianum, Croton macrostachyus, Rhus glutinosa, Celtis africana, Vachellia polyacantha, and Millettia ferruginea* were the dominant species of the tree layer of the community. In the shrub layer, *Capparis tomentosa, Rumex nervosus, Senna singueana,* and *Dombeya torrida* were the dominant species.

### Community 3, *Euclea racemosa—Maytenus senegalensis* community type

This community is distributed between the altitudinal ranges of 2030 and 2133 m a.s.l. It is represented by 26 plots consisting of 59 species; out of which 28 species are shared with community one and 27 are shared with community two. *Maytenus senegalensis* and *Euclea racemosa* are the characteristic species in this community. Dominant tree and shrub plant species found in this community include *Rhus glutinosa, Vachellia bussei, Ficus sycomorus, Rhus vulgaris, Carissa spinarum, Euclea racemosa, Premna schimperi, Maytenus senegalensis, Calpurnia aurea, Senna singueana, Otostegia integrifolia,* and *Vachellia brevispica*.

### Anthropogenic disturbance in three communities

**Fig. 6:**
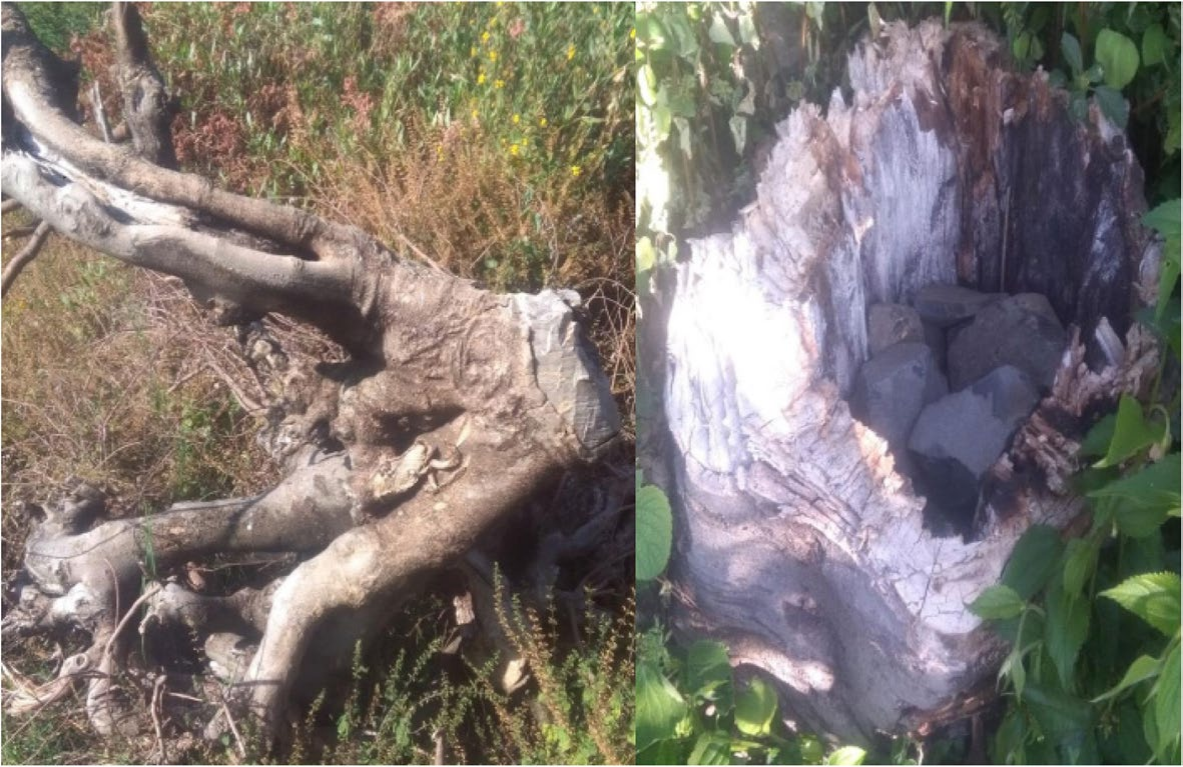
Cutting of *Celtis africana* for timber production (Photo by Yitayih Dagne, 2021)

**Fig. 7:**
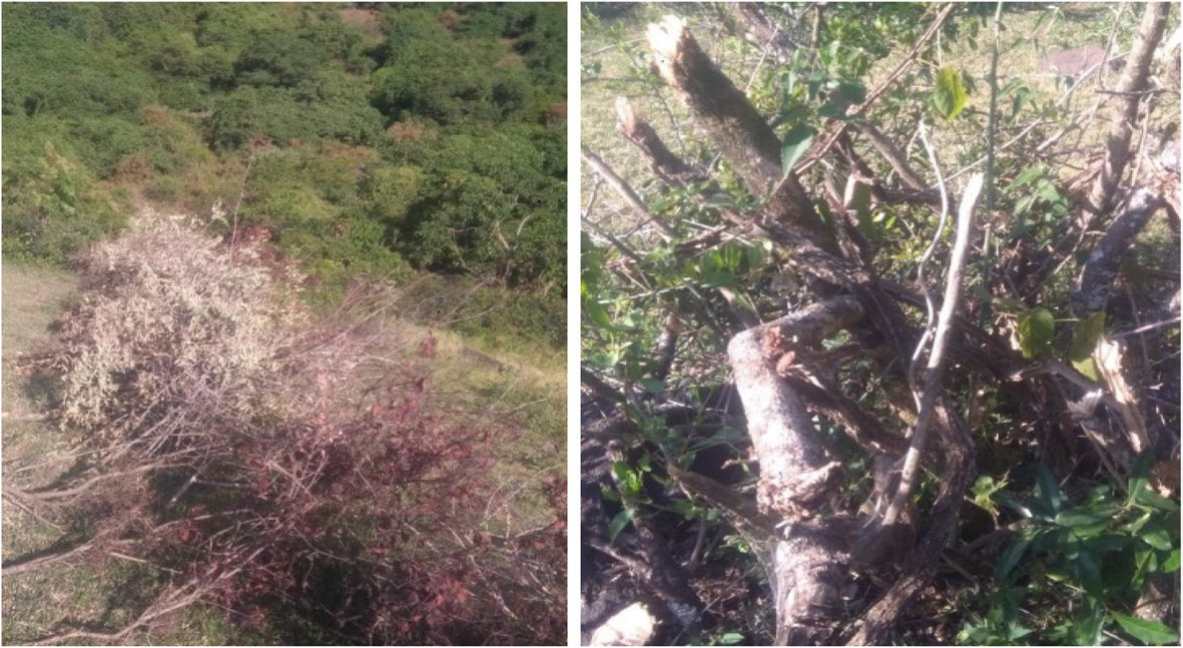
Cutting of *Maytenus senegalensis* for fencing and firewood (Photo by Yitayih Dagne, 2021)

**Fig. 8:**
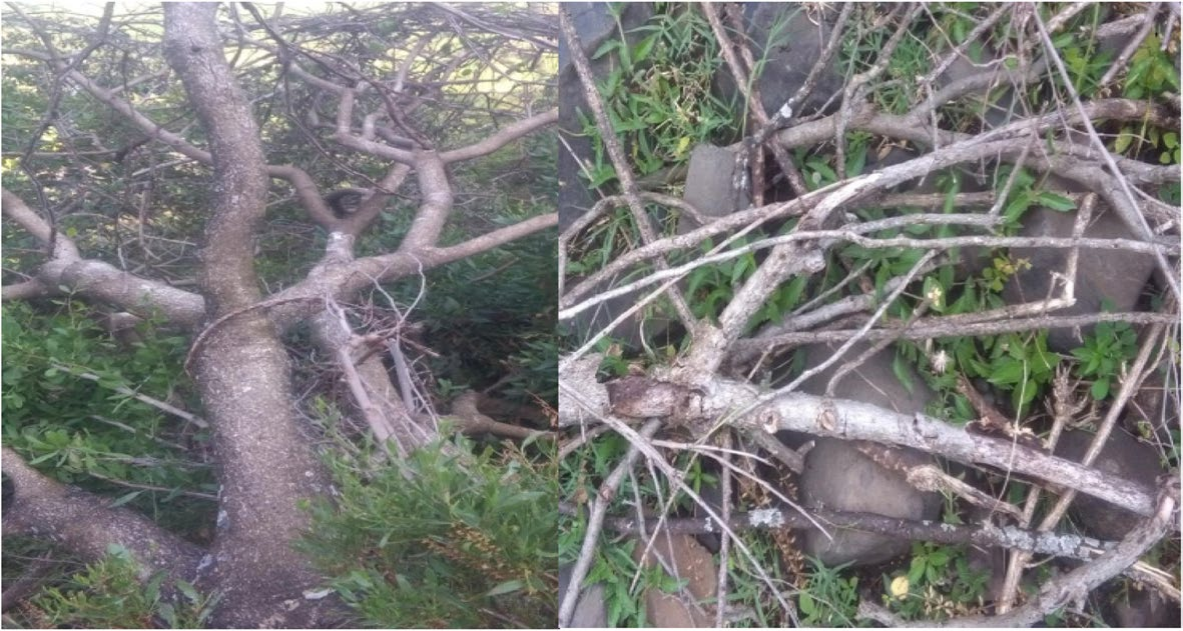
Cutting of *Croton macrostachus* for firewood (Photo by Yitayih Dagne, 2021)

The analysis of data shows that the forest structural attributes such as species diversity, richness, evenness, and canopy cover become different between each community type. The level of anthropogenic disturbance in the three plant communities varies from a maximum mean score of 8.5 for community type 3 for firewood collection, timber, charcoal**,** grazing, and others to a minimum mean score of 7.2 for community type 2 (Table 6). In all community types, there is an anthropogenic disturbance for firewood, timber, grazing, charcoal, and others (Figs. 6, 7 and 8). Community type 3 were ranked as highly disturbed in all categories and showed great disturbance than the other communities because of its exposure to cultivation, grazing, and human settlement, but plant community type 1 showed less level of anthropogenic disturbance, and plots 5, 7, 9, and 13 in community types 1 and 3 did not show any sign of anthropogenic disturbance.

**Table 6:** The mean value of anthropogenic disturbances in each community type

Disturbance Variable	Disturbances Score					
	**C**_**1**_		**C**_**2**_		**C**_**3**_	
	**mean**	**SD**	**mean**	**SD**	**Mean**	**SD**
**Fire wood**	1.79	0.83	1.75	0.46	1.85	0.83
**Timber**	1.54	0.78	1.75	0.71	1.58	0.9
**Fodder**	1.46	0.83	1.63	0.74	1.46	0.76
**Charcoal**	0.92	0.72	0.88	0.35	0.92	0.48
**Grazing**	1.63	1.21	2.5	0.76	1.46	0.95

One way ANOVA showed that there was a statistically significant difference among plant communities in anthropogenic disturbances for firewood collection, timber, charcoal**,** grazing, and others (Table 7).

**Table 7:** The significance of disturbances between communities

**ANOVA **Value					
	Sum of squares	df	Mean square	F	Sig.
Between groups	1.737	4	.434	6.455	.008
Within groups	.673	10	.067		
Total	2.410	14			

### Relationship between community types and environmental factors

The performed DCA to select the best ordination technique showed that the gradient length is 3.35 is higher than 3, So CCA was approved to be an appropriate ordination for testing the vegetation and environmental variables relationships. In this study, the longest axis of DCA for Guard forest data set was 3.35, indicating that the data were heterogeneous as shown in (Table 8). Therefore, an analysis with the unimodal response model is preferred and the Canonical correspondence analysis (CCA) ordination method is used for this analysis for testing the community type and environmental variable relationships.

**Table 8:** Detrended correspondence analysis result of Guard forest

	DCA1	DCA2	DCA3	DCA4
Eigenvalues	0.4010	0.3066	0.2693	0.2231
Decorana values	0.4157	0.2970	0.2311	0.1819
Axis lengths	3.3547	2.7219	2.4494	2.2661

A total of 16 different environmental factors were measured, but the most influential environmental factors that significantly affect the distribution of plants in the Guard forest were altitude, pH, BD, slope, and charcoal, and others were less significant (Table 9).

**Table 9:** Most influential environmental factors that affect the distribution of plants in Guard forest

Variable	Df	Sums of Sqs.	Mean Sqs.	F. Model	R^2^	Pr(> F)
Altitude	1	0.8548	0.85476	3.6653	0.05287	0.001 ***
PH	1	0.4622	0.46218	1.9819	0.02859	0.026 *
BD	1	0.7775	0.77752	3.3341	0.04809	0.001 ***
PD	1	0.8409	0.84094	3.6060	0.05201	0.002 **
Slope	1	0.4724	0.47244	2.0259	0.02922	0.019 *
Charcoal	1	0.4388	0.43883	1.8818	0.02714	0.038 *
Residuals	42	9.7945	0.23320	0.60579		
Total	57	16.1682	1.00000			

Signif. codes: ‘***’ 0.001,‘**’ 0.01,‘*’ 0.05

**Fig. 9:**
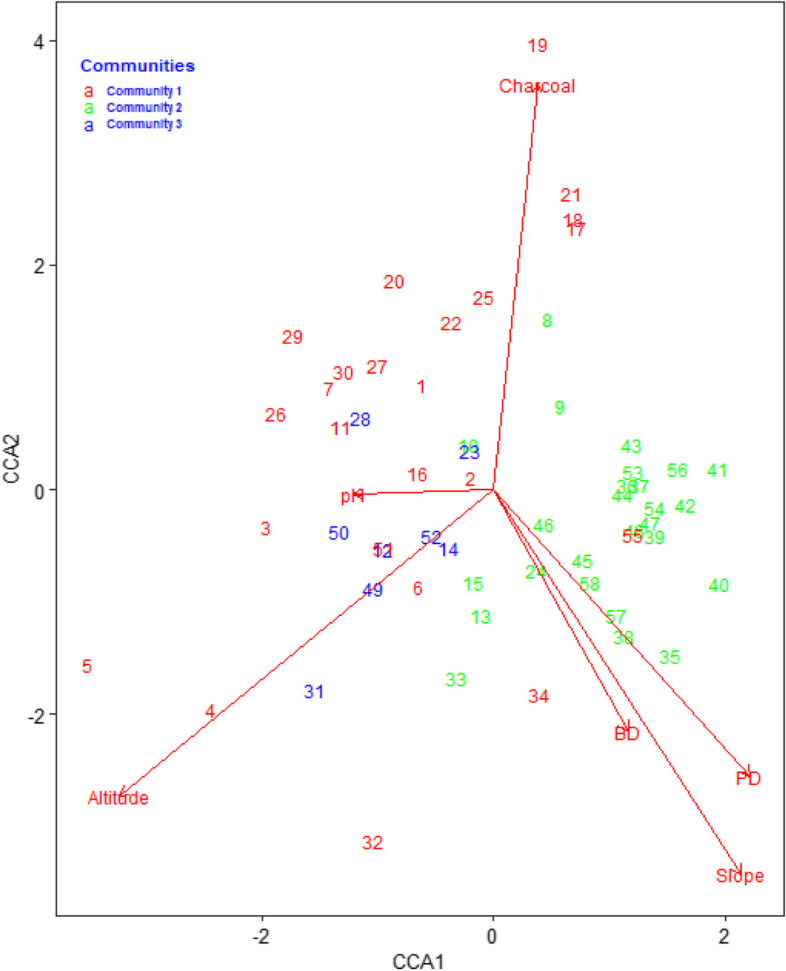
Canonical Correspondence Analysis (CCA) ordination graph significant environmental variables (*p* < 0.05) and the plant community in the study area. The arrows in the diagram stand for the environmental factors, the length of each arrow indicates the contribution of the factor to ordination axes, the numbers refer to quadrat number, and the angle between the arrows and he axes indicates the correlation between the variable and the ordination axe

The cumulative proportion of variance explained by the first six axes of the joint plot in the constrained biplot at Guard forest was 94%. From the cumulative proportion of variance explained by the first six axes, the proportion of variation explained by the first two axes at Guard forest is 54% (Table 10, Fig. 9).

**Table 10:** Biplot scores for constraining variables

Variables	CCA1	CCA2	CCA3	CCA4	CCA5	CCA6
Altitude	-0.579	-0.473	0.43	-0.21	0.053	-0.44
pH	-0.218	-0.0061	0.66	0.25	-0.650	0.18
BD	0.208	-0.3843	0.44	-0.54	-0.520	0.23
PD	0.397	-0.4561	0.77	-0.14	0.082	-0.14
Slope	0.383	-0.6117	-0.15	0.11	-0.416	-0.52
Eigen value	0.2736	0.1825	0.1220	0.1159	0.09807	0.04967
Proportion Explained	0.3250	0.2169	0.1449	0.130	0.01651	0.05901
Cumulative proportion	0.3250	0.5419	0.6868	0.8245	0.94099	1.00000

Constraining variables highly correlated with axis one contributed more eigenvalues, indicating that more variation was explained by these constraining variables. The eigenvalue for axis one was higher than others. Consequently, the first two axes are sufficient to reflect the relationship between community type and environmental factors (Fig. 9).

## Discussion

### Floristic composition

Guard forest has high species richness similar to other dry Afromontane forests. Besides, *Olea europaea* subsp. *cuspidata*, *Juniperus procera,* and *Podocarpus falcatus* are indicator species to the dry Afromontane forest [2]. These species are not found in the Guard forest but they are located in the church and private home garden near to the forest, which could be the effect of anthropogenic disturbances in the study forest. The dry Afromontane vegetation is under threat due to due to human-induced pressure such as agricultural expansion, overexploitation, and settlement[2]. Thus, special attention should be given to protect these indicator species from dry Afromontane forest.

Different study has been conducted in different parts of the country to determine the floristic composition and species diversity of Afromontane forests, but it is difficult to directly compare with other similar studies due to the difference in forest size, topographic location, sampling technique, sample size and the degree of human and livestock disturbances, however, the overall species richness of a given forest can give a general impression on the diversity of study area [21]. The total floristic composition of Guard forest was 137 species and 83 woody plant species recorded in this study area that can relate to other dry evergreen Afromontane forests in Ethiopia, For instance, from Masha forest [21] identified 130 species; Gelawoldie community forest [10] identifies 59 woody plant species and from Debark District [22] identifies 55 woody species. This study shows that the Guard forest has relatively good species composition and richness compared to other dry Afromontane forests.

The distribution of species across the families was not even about (27%) of the species recorded from the two most dominant families. Fabaceae, Asteraceae, Acanthaceae, Euphorbiaceae**,** Poaceae**,** Lamiaceae, Solanaceae, and Moraceae are among the richest plant families. These plant families are also identified as the top dominant plant families in the Flora of Ethiopia and Eritrea [5]. Fabaceae was the most dominant family with 20 species (15%), followed by Asteraceae 16 species (12%), and Lamiaceae 7 species (5%). The dominance of Fabaceae and Asteraceae was confirmed by other similar studies [10, 22, 23] in contrast to those studies, Fabaceae is higher than Asteraceae. The dominance of Fabaceae and Asteraceae in the present study may indicate that the forest experienced disturbance. The success of Fabaceae in dominating disturbed habitats is ascribed to the ability of the species to fix atmospheric nitrogen, thus allowing them to grow in nutrient-poor soils [24]. Likewise, plants of Asteraceae are often ruderal and normally prefer open and disturbed lands to grow [25].

Regarding the habits of the species, all plant habits were identified of which 35.04% were recorded as herbs, followed by a tree (30.65%), shrub (26.28%, and climber (8.03%) respectively. Herbs contain the highest number of species compared with other growth forms collected in the study area. This result is in agreement with a similar pattern of dominance of herbs in the forest [26]. The highest representation of herbs in Guard forest compared to other growth forms might be due to high anthropogenic disturbance during the cutting of trees and shrubs for firewood, timber, and charcoal; low density, and frequency of shrub and tree species**.**

The result of endemic species shows that about 10.22% are endemic to Ethiopia and Eritrea, this is in agreement with the study of [27] confirming that the Afromontane forest of Ethiopia contains 10–15% endemic plant species and is also related with similar studies of [28] recorded 14% [23] record 10.6% Endemic plant species from the total floristic composition. Some of endemic species are vulnerable and near threatened to conserve those species, the community shall be wisely using those species by cultivating them in their home garden and use an alternative species. The concerned bodies also create awareness for the local communities on resource management.

### Plant community types and species diversity

The Shannon–Wiener diversity index of Guard forest varies between 3.03 and 3.82 with the mean value of 3.39. This result shows that the study area has medium species diversity which is lower than [28, 29] but it’s better than [30, 31] and the species diversity varies between each community type. The medium Shannon–Wiener diversity index of a forest might be due to continuous cutting of trees for firewood and timber, exposure for grazing, removing of shrubs and trees for expansion of agriculture, and collection of fodder. The variation of diversity value between community types might be due to the location of each community type on the human settlements, cultivation area, and exposure to landslides and flooding.

### The relationship between plant community types and environmental factors

Different environmental and anthropogenic disturbance factors greatly affecting the diversity and distribution of plant species. Altitude plays an important role in determining species diversity and abundance [21]. Similarly, the analysis of the relationship between plant communities and environmental factors using ordination techniques indicated that the pattern of plant species distribution was significantly influenced by environmental factors such as altitude, pH, BD, slope, and charcoal while others were less significant at higher altitudes the distribution of species increased and this might be due to the location of human settlement is more common at lower altitude than higher altitude that decreases the cutting of tree than lower altitude. A similar study by [16] confirms that altitudinal variation and anthropogenic disturbances have a significant effect on the structure and diversity of the forest. In the natural environment, the pH of the soil has an enormous influence on soil biogeochemical processes described as the main factor that influences many soil biological, chemical, and physical properties and processes that affect plant growth and productivity [32].

## Conclusion

The result of the floristic composition of Guard forest shows that the area has good species diversity and richness. Of the total species, 14(10.22%) are endemic and this number fulfills the expected proportion of endemic species from dry evergreen Afromontane forests. The presence of more endemic plant species in the forest show the potential of the area to support useful species**.** Three plant communities were identified in this study. Among all investigated environmental factors and anthropogenic disturbances, altitude, pH, BD, slope, and charcoal were found to significantly explain the variation in species composition and community formation in the study area. The forest is ecologically, economically, and culturally essential for the local people living around the forest due to its importance to the community, and some of the important characteristics of species like *Olea europaea* subsp. *cuspidata*, *Juniperus procera,* and *Podocarpus falcatus* are not found in the forest and others are rare in their existence, such species require urgent conservation measures that will enhance healthy regeneration and guarantee sustainable use of these species.

### Supplementary Information

**Additional file 1.** 

**Additional file 2.**

**Additional file 3.**

## Data Availability

The data used to support the findings of this study are included within the article and its supplementary information files.

## Abbreviations

CCA: Canonical Correspondence Analysis
DCA: Detrended Correspondence Analysis
