# Supplementary material for: Floristic composition and plant community distribution along environmental gradients in Guard dry Afromontane forest of Northwestern Ethiopia
Source: BMC Ecol Evol. 2023 Aug 28;23:43. doi: 10.1186/s12862-023-02154-6 (PMC10463663; doi:10.1186/s12862-023-02154-6)
Supplement: Supplementary file 1 — Additional file 1. [file 12862_2023_2154_MOESM1_ESM.docx]

Additional file 1: Sample plot corresponding to environmental factors.

| Transect | plot | Altitude | Latitude | Longitude | Slope (%) |
| --- | --- | --- | --- | --- | --- |
| 1 | 1 | 2045 | UTM1139744 | 37P0417247 | 10 |
|  | 2 | 2049 | UTM1139743 | 37P0417247 | 30 |
|  | 3 | 2109 | UTM1139826 | 37P0417157 | 20 |
|  | 4 | 2153 | UTM1139818 | 37P0417116 | 10 |
| 2 | 5 | 2178 | UTM1139793 | 37P0417071 | 10 |
|  | 6 | 2136 | UTM1139716 | 37P0417069 | 10 |
|  | 7 | 2098 | UTM1139666 | 37P0417107 | 5 |
|  | 8 | 2041 | UTM1139612 | 37P0417165 | 5 |
|  | 9 | 2049 | UTM1139542 | 37P0417119 | 5 |
| 3 | 10 | 2058 | UTM1139558 | 37P0417082 | 10 |
|  | 11 | 2097 | UTM1139614 | 37P0417070 | 10 |
|  | 12 | 2134 | UTM1139653 | 37P0417035 | 10 |
|  | 13 | 2115 | UTM1139584 | 37P0417004 | 20 |
|  | 14 | 2097 | UTM1139566 | 37P0417014 | 20 |
|  | 15 | 2068 | UTM1139514 | 37P0417023 | 30 |
| 4 | 16 | 2124 | UTM1139514 | 37P0417023 | 40 |
|  | 17 | 2112 | UTM1139471 | 37P0417076 | 20 |
|  | 18 | 2103 | UTM1138400 | 37P0416416 | 10 |
|  | 19 | 2093 | UTM1138400 | 37P0416419 | 10 |
|  | 20 | 2083 | UTM1138850 | 37P0416922 | 5 |
|  | 21 | 2062 | UTM1139100 | 37P0417029 | 5 |
|  | 22 | 2051 | UTM1139242 | 37P0417001 | 5 |
|  | 23 | 2044 | UTM1139312 | 37P0417038 | 5 |
| 5 | 24 | 2033 | UTM1139362 | 37P0417070 | 5 |
|  | 25 | 2040 | UTM1139363 | 37P0417070 | 5 |
|  | 26 | 2057 | UTM1139329 | 37P0417037 | 5 |
|  | 27 | 2072 | UTM1139246 | 37P0416918 | 10 |
|  | 28 | 2087 | UTM1139230 | 37P0416902 | 10 |
|  | 29 | 2111 | UTM1139236 | 37P0416874 | 20 |
|  | 30 | 2148 | UTM1139161 | 37P0416830 | 20 |
|  | 31 | 2211 | UTM1139248 | 37P0416793 | 30 |
| 6 | 32 | 2205 | UTM1139340 | 37P0416773 | 40 |
|  | 33 | 2155 | UTM1139584 | 37P0417004 | 40 |
|  | 34 | 2129 | UTM1139566 | 37P0417014 | 40 |
|  | 35 | 2064 | UTM1139440 | 37P0416776 | 40 |
|  | 36 | 2056 | UTM1139282 | 37P0416002 | 40 |
|  | 37 | 2042 | UTM1139167 | 37P0416016 | 40 |
| 7 | 38 | 2164 | UTM1138801 | 37P0416718 | 43 |
|  | 39 | 2154 | UTM1138792 | 37P0416745 | 30 |
|  | 40 | 2083 | UTM1138746 | 37P0416828 | 30 |
|  | 41 | 2023 | UTM1138604 | 37P0416852 | 20 |
| 8 | 42 | 2014 | UTM1138580 | 37P0416852 | 10 |
|  | 43 | 2030 | UTM1138501 | 37P0416637 | 20 |
|  | 44 | 2034 | UTM1138567 | 37P0416718 | 30 |
|  | 45 | 2059 | UTM1138617 | 37P0416693 | 30 |
| 9 | 46 | 2069 | UTM1138639 | 37P0416680 | 30 |
|  | 47 | 2099 | UTM1138680 | 37P0416647 | 20 |
|  | 48 | 2131 | UTM1138740 | 37P0416650 | 10 |
|  | 49 | 2144 | UTM1138749 | 37P0416648 | 10 |
|  | 50 | 2159 | UTM1138744 | 37P0416569 | 5 |
| 10 | 51 | 2123 | UTM1138703 | 37P0416566 | 10 |
|  | 52 | 2103 | UTM1138663 | 37P0416563 | 20 |
|  | 53 | 2074 | UTM1138610 | 37P0416594 | 20 |
|  | 54 | 2060 | UTM1138556 | 37P0416617 | 30 |
|  | 55 | 2054 | UTM1138531 | 37P0416506 | 30 |
| 11 | 56 | 2041 | UTM1138454 | 37P0416402 | 10 |
|  | 57 | 2055 | UTM1138533 | 37P0416504 | 20 |
|  | 58 | 2073 | UTM1138577 | 37P0416472 | 30 |
