# Supplementary material for: Floristic composition and plant community distribution along environmental gradients in Guard dry Afromontane forest of Northwestern Ethiopia
Source: BMC Ecol Evol. 2023 Aug 28;23:43. doi: 10.1186/s12862-023-02154-6 (PMC10463663; doi:10.1186/s12862-023-02154-6)
Supplement: Supplementary file 2 — Additional file 2. [file 12862_2023_2154_MOESM2_ESM.docx]

Additional file 2: Value of disturbances on each plot (all types of disturbances were ranked into relatively absent (no disturbance), score 0, low (1), medium (2), and high (3)for each level of disturbance)

| Plot No | Disturbances type | | | | |
| --- | --- | --- | --- | --- | --- |
|  | Fire wood | Timber | Fodder | Charcoal | Grazing |
| 1 | 3 | 3 | 2 | 1 | 0 |
| 2 | 2 | 1 | 2 | 1 | 0 |
| 3 | 2 | 2 | 1 | 1 | 0 |
| 4 | 3 | 3 | 0 | 1 | 0 |
| 5 | 0 | 0 | 0 | 0 | 0 |
| 6 | 1 | 1 | 1 | 1 | 0 |
| 7 | 0 | 0 | 0 | 0 | 0 |
| 8 | 1 | 0 | 1 | 1 | 0 |
| 9 | 0 | 0 | 0 | 0 | 0 |
| 10 | 2 | 1 | 0 | 1 | 0 |
| 11 | 3 | 2 | 3 | 1 | 3 |
| 12 | 3 | 2 | 1 | 1 | 3 |
| 13 | 0 | 0 | 0 | 0 | 0 |
| 14 | 2 | 2 | 3 | 1 | 3 |
| 15 | 2 | 2 | 0 | 0 | 0 |
| 16 | 1 | 2 | 1 | 1 | 3 |
| 17 | 3 | 2 | 2 | 2 | 3 |
| 18 | 3 | 2 | 1 | 2 | 2 |
| 19 | 3 | 3 | 3 | 3 | 3 |
| 20 | 1 | 2 | 1 | 1 | 3 |
| 21 | 3 | 3 | 3 | 1 | 3 |
| 22 | 3 | 3 | 3 | 1 | 3 |
| 23 | 3 | 2 | 1 | 1 | 3 |
| 24 | 3 | 3 | 3 | 0 | 3 |
| 25 | 3 | 3 | 3 | 1 | 3 |
| 26 | 3 | 3 | 3 | 0 | 3 |
| 27 | 3 | 3 | 3 | 1 | 3 |
| 28 | 3 | 3 | 3 | 1 | 3 |
| 29 | 3 | 3 | 3 | 1 | 3 |
| 30 | 3 | 3 | 3 | 1 | 3 |
| 31 | 3 | 2 | 2 | 1 | 3 |
| 32 | 2 | 1 | 1 | 0 | 3 |
| 33 | 3 | 1 | 1 | 1 | 3 |
| 34 | 1 | 2 | 2 | 0 | 1 |
| 35 | 2 | 1 | 1 | 1 | 2 |
| 36 | 2 | 1 | 1 | 1 | 2 |
| 37 | 2 | 2 | 2 | 1 | 2 |
| 38 | 3 | 3 | 3 | 1 | 3 |
| 39 | 3 | 3 | 3 | 1 | 3 |
| 40 | 2 | 2 | 2 | 0 | 2 |
| 41 | 2 | 2 | 1 | 1 | 2 |
| 42 | 3 | 2 | 2 | 0 | 3 |
| 43 | 1 | 1 | 1 | 1 | 1 |
| 44 | 1 | 1 | 1 | 0 | 1 |
| 45 | 2 | 2 | 2 | 1 | 2 |
| 46 | 2 | 2 | 2 | 1 | 2 |
| 47 | 1 | 1 | 1 | 1 | 1 |
| 48 | 1 | 1 | 2 | 1 | 1 |
| 49 | 2 | 3 | 1 | 0 | 2 |
| 50 | 3 | 3 | 2 | 1 | 2 |
| 51 | 2 | 1 | 2 | 1 | 1 |
| 52 | 1 | 1 | 1 | 1 | 1 |
| 53 | 0 | 1 | 1 | 1 | 0 |
| 54 | 2 | 1 | 1 | 1 | 2 |
| 55 | 2 | 1 | 1 | 0 | 2 |
| 56 | 1 | 1 | 1 | 1 | 1 |
| 57 | 0 | 1 | 1 | 0 | 0 |
| 58 | 2 | 3 | 2 | 1 | 2 |
