# Supplementary material for: Floristic composition and plant community distribution along environmental gradients in Guard dry Afromontane forest of Northwestern Ethiopia
Source: BMC Ecol Evol. 2023 Aug 28;23:43. doi: 10.1186/s12862-023-02154-6 (PMC10463663; doi:10.1186/s12862-023-02154-6)
Supplement: Supplementary file 3 — Additional file 3. [file 12862_2023_2154_MOESM3_ESM.docx]

Additional file 3: The measure of soil property in Guard forest

(EC= electrical conductivity, BD=bulk density, PD= particle density, MC=moisture content, OM=organic matter).

| **plot** | **pH** | **EC** | **BD** | **PD** | **MC** | **OM** | **Sand** | **clay** | **Silt** | **Texture** |
| --- | --- | --- | --- | --- | --- | --- | --- | --- | --- | --- |
| 1 | 6.3 | 622 | 0.96 | 1.42 | 11.62 | 10.1 | 30 | 32 | 38 | Clay Loam |
| 2 | 6.3 | 622 | 0.96 | 1.42 | 11.62 | 10.1 | 30 | 32 | 38 | Clay Loam |
| 3 | 6.3 | 622 | 0.96 | 1.42 | 11.62 | 10.1 | 30 | 32 | 38 | Clay Loam |
| 4 | 6.7 | 172 | 1.12 | 2.07 | 9.19 | 8.28 | 35 | 23 | 42 | Loam |
| 5 | 6.7 | 172 | 1.12 | 2.07 | 9.19 | 8.28 | 35 | 23 | 42 | Loam |
| 6 | 6.1 | 697 | 0.76 | 1.92 | 29.5 | 12.8 | 72 | 4 | 24 | Sandy Loam |
| 7 | 6.1 | 697 | 0.76 | 1.92 | 29.5 | 12.8 | 72 | 4 | 24 | Sandy Loam |
| 8 | 6.1 | 697 | 0.76 | 1.92 | 29.5 | 12.8 | 72 | 4 | 24 | Sandy Loam |
| 9 | 6.3 | 622 | 0.96 | 1.42 | 11.62 | 10.1 | 30 | 32 | 38 | Clay Loam |
| 10 | 6.3 | 622 | 0.96 | 1.42 | 11.62 | 10.1 | 30 | 32 | 38 | Clay Loam |
| 11 | 6.8 | 655 | 0.82 | 1.7 | 41.4 | 17.5 | 76 | 16 | 8 | Sandy Loam |
| 12 | 6.9 | 458 | 1.19 | 2.29 | 15.3 | 8.83 | 54 | 24 | 22 | Sand Clay Loam |
| 13 | 6.9 | 458 | 1.19 | 2.29 | 15.3 | 8.83 | 54 | 24 | 22 | Sand Clay Loam |
| 14 | 6.7 | 172 | 1.12 | 2.07 | 9.19 | 8.28 | 35 | 23 | 42 | Loam |
| 15 | 6.7 | 172 | 1.12 | 2.07 | 9.19 | 8.28 | 35 | 23 | 42 | Loam |
| 16 | 6.8 | 655 | 0.82 | 1.7 | 41.4 | 17.5 | 76 | 16 | 8 | Sandy Loam |
| 17 | 6.5 | 440 | 0.96 | 2.47 | 7.53 | 8.42 | 51 | 26 | 23 | Sand Clay Loam |
| 18 | 6.3 | 305 | 0.73 | 1.96 | 10.6 | 7.48 | 27 | 45 | 28 | Clay |
| 19 | 6.8 | 552 | 1.19 | 2.29 | 6.89 | 4.61 | 48 | 24 | 28 | Sand Clay Loam |
| 20 | 6.3 | 305 | 0.73 | 1.96 | 10.6 | 7.48 | 27 | 45 | 28 | Clay |
| 21 | 6.9 | 458 | 1.19 | 2.29 | 15.3 | 8.83 | 54 | 24 | 22 | Sand Clay Loam |
| 22 | 6.4 | 895 | 0.92 | 2.06 | 11.8 | 9.68 | 32 | 34 | 34 | Clay Loam |
| 23 | 6.4 | 895 | 0.92 | 2.06 | 11.8 | 9.68 | 32 | 34 | 34 | Clay Loam |
| 24 | 6.8 | 655 | 0.82 | 1.7 | 41.4 | 17.5 | 76 | 16 | 8 | Sandy Loam |
| 25 | 6.8 | 655 | 0.82 | 1.7 | 41.4 | 17.5 | 76 | 16 | 8 | Sandy Loam |
| 26 | 6.8 | 655 | 0.82 | 1.7 | 41.4 | 17.5 | 76 | 16 | 8 | Sandy Loam |
| 27 | 6.9 | 458 | 0.86 | 1.98 | 15.3 | 8.83 | 54 | 24 | 22 | Sand Clay Loam |
| 28 | 6.8 | 655 | 0.82 | 1.7 | 41.4 | 17.5 | 76 | 16 | 8 | Sandy Loam |
| 29 | 6.8 | 655 | 0.82 | 1.7 | 41.4 | 17.5 | 76 | 16 | 8 | Sandy Loam |
| 30 | 6.8 | 655 | 0.82 | 1.7 | 41.4 | 17.5 | 76 | 16 | 8 | Sandy Loam |
| 31 | 6.6 | 186 | 1.13 | 3.33 | 7.95 | 4.49 | 46 | 26 | 28 | Sand Clay Loam |
| 32 | 6.6 | 186 | 1.13 | 3.33 | 7.95 | 4.49 | 46 | 26 | 28 | Sand Clay Loam |
| 33 | 6.6 | 186 | 1.13 | 3.33 | 7.95 | 4.49 | 46 | 26 | 28 | Sand Clay Loam |
| 34 | 6.5 | 324 | 1.13 | 2.35 | 8.53 | 8.28 | 46 | 19 | 35 | Loam |
| 35 | 6.5 | 324 | 1.13 | 2.35 | 8.53 | 8.28 | 46 | 19 | 35 | Loam |
| 36 | 6.5 | 324 | 1.13 | 2.35 | 8.53 | 8.28 | 46 | 19 | 35 | Loam |
| 37 | 6.1 | 697 | 0.76 | 1.92 | 29.5 | 12.8 | 72 | 4 | 24 | Sandy Loam |
| 38 | 6.8 | 552 | 1.13 | 3.33 | 6.89 | 4.61 | 48 | 24 | 28 | Sand Clay Loam |
| 39 | 6.8 | 552 | 1.13 | 3.33 | 6.89 | 4.61 | 48 | 24 | 28 | Sand Clay Loam |
| 40 | 6.8 | 552 | 1.13 | 3.33 | 6.89 | 4.61 | 48 | 24 | 28 | Sand Clay Loam |
| 41 | 6.8 | 552 | 1.13 | 3.33 | 6.89 | 4.61 | 48 | 24 | 28 | Sand Clay Loam |
| 42 | 6.5 | 440 | 0.96 | 2.47 | 7.53 | 8.42 | 51 | 26 | 23 | Sand Clay Loam |
| 43 | 6.3 | 305 | 0.73 | 1.96 | 10.6 | 7.48 | 27 | 45 | 28 | Clay |
| 44 | 6.5 | 440 | 0.96 | 2.47 | 7.53 | 8.42 | 51 | 26 | 23 | Sand Clay Loam |
| 45 | 6.3 | 622 | 0.96 | 1.42 | 11.62 | 10.1 | 30 | 32 | 38 | Clay Loam |
| 46 | 6.3 | 622 | 0.96 | 1.42 | 11.62 | 10.1 | 30 | 32 | 38 | Clay Loam |
| 47 | 6.6 | 186 | 1.13 | 3.33 | 7.95 | 4.48 | 46 | 26 | 28 | Sand Clay Loam |
| 48 | 6.6 | 186 | 1.13 | 3.33 | 7.95 | 4.48 | 46 | 26 | 28 | Sand Clay Loam |
| 49 | 6.1 | 697 | 0.76 | 1.92 | 29.5 | 12.8 | 72 | 4 | 24 | Sandy Loam |
| 50 | 6.1 | 697 | 0.76 | 1.92 | 29.5 | 12.8 | 72 | 4 | 24 | Sandy Loam |
| 51 | 6.3 | 305 | 0.73 | 1.96 | 10.6 | 7.48 | 27 | 45 | 28 | Clay |
| 52 | 6.3 | 305 | 0.73 | 1.96 | 10.6 | 7.48 | 27 | 45 | 28 | Clay |
| 53 | 6.1 | 697 | 0.76 | 1.92 | 29.5 | 12.8 | 72 | 4 | 24 | Sandy Loam |
| 54 | 6.1 | 697 | 0.76 | 1.92 | 29.5 | 12.8 | 72 | 4 | 24 | Sandy Loam |
| 55 | 6.5 | 324 | 1.13 | 2.35 | 8.53 | 8.28 | 46 | 19 | 35 | Loam |
| 56 | 6.5 | 324 | 1.13 | 2.35 | 8.53 | 8.28 | 46 | 19 | 35 | Loam |
| 57 | 6.3 | 305 | 0.73 | 1.96 | 10.6 | 7.48 | 27 | 45 | 28 | Clay |
| 58 | 6.3 | 305 | 0.73 | 1.96 | 10.6 | 7.48 | 27 | 45 | 28 | Clay |
